# Diagnostics of Ebola virus

**DOI:** 10.3389/fpubh.2023.1123024

**Published:** 2023-02-23

**Authors:** Aurora Bettini, Daniele Lapa, Anna Rosa Garbuglia

**Affiliations:** Laboratory of Virology, National Institute for Infectious Diseases Lazzaro Spallanzani (IRCCS), Rome, Italy

**Keywords:** Ebola, diagnostics, outbreak, molecular test, rapid diagnostic test (RDT), real-time

## Abstract

Ebola is a highly pathogenic virus, which in humans reaches a mortality rate above 50%. Due to a lack of laboratories in territories where Ebola viruses are endemic and the limited number of surveillance programmes, tests for the confirmation of suspected cases of Ebola are often performed in Reference Laboratories. While this provides guarantees regarding the accuracy of results, the shipment of samples to a centralized facility where the diagnostic test can be performed and the time required to achieve the results takes several days, which increases costs and entails delays in the isolation of positive subjects and therapeutic intervention with negative consequences both for patients and the community. Molecular tests have been the most frequently used tool in Ebola diagnosis in recent outbreaks. One of the most commonly used molecular tests is the Real-Star Altona, which targets a conserved area of the L gene. This assay showed different sensitivities depending on the Ebola virus: 471 copies/mL (EBOV) and 2871 copies/ml (SUDAN virus). The Cepheid system also showed good sensitivity (232 copies/mL). The LAMP platform is very promising because, being an isothermal reaction, it does not require high-precision instrumentation and can be considered a Point of Care (PoC) tool. Its analytical sensitivity is 1 copy/reaction. However, since data from real life studies are not yet available, it is premature to give any indications on its feasibility. Moreover, in November 2014, the WHO recommended the development of rapid diagnostic tests (RDT) according to ASSURED criteria. Several RDT assays have since been produced, most of which are rapid tests based on the search for antibody anti-Ebola viral proteins with immunochromatographic methods. Several viral antigens are used for this purpose: VP40, NP and GP. These assays show different sensitivities according to the protein used: VP40 57.4–93.1%, GP 53–88.9% and 85% for NP compared to reference molecular assays. From these results, it can be deduced that no RDT reaches the 99% sensitivity recommended by the WHO and therefore any RDT negative results in suspected cases should be confirmed with a molecular test.

## Introduction

Ebola viruses represent one of the most dangerous pathogens for humans with a mortality rate of Zaire virus (one of the five Ebola viruses which can infect humans) exceeding 50% (1).

Since 1976 (the year of the first Ebola virus isolation), several Ebola outbreaks and epidemics have taken place in endemic areas. In 2014–2015, an outbreak which began in a remote village in Guinea, Guéckédou, appears to be the largest outbreak with 28,646 confirmed cases and 11,323 deaths (2, 3). Given that the transmission of the disease mainly occurs through person-to-person contact and with infected material (burial, body fluids), the lack of rapid diagnosis of suspected cases facilitates the rapid spread of the infection. Furthermore, the various epidemics occurred in countries with very weak health systems and characterized by few health care workers and limited infrastructures (4, 5).

Due to a lack of high containment laboratories in territories where Ebola viruses are endemic and the limited number of surveillance programmes, tests for the confirmation of suspected cases of Ebola are often performed in Reference Laboratories.

While this provides guarantees regarding the accuracy of results, it takes time to ship samples to a centralized facility where the diagnostic test can be performed and further time to obtain results. These diagnostic procedures increase costs and entail delays in the isolation of positive subjects and therapeutic intervention with negative consequences both for patients and the community.

Molecular tests have been the most frequently used for Ebola diagnosis in the last outbreaks. However, in November 2014, the WHO (World Health Organization) recommended the development of rapid diagnostic tests (RDT) that could be used by non-highly specialized personnel and without the nucleic acid extraction amplification step.^1^

In addition, given that the clinical symptoms of Ebola are similar to those caused by other pathogens such as Yellow Fever virus (YFV), Rift Valley Fever virus (RVFV), Lassa virus (LASV), O'Nyong-Nyong virus (ONNV), rickettsias, Borrelia spp, *Coxiella burnetii*, and malaria, it is important to use diagnostic methods for differential diagnosis in order to administer a correct therapy and for proper patient management (6).

Despite the efforts made in the last decade in R&D regarding a PoC set-up, including microfluidics that use low-cost materials and which can be used on a wide scale in PoC units, the accuracy of current assays are not yet optimal and often they need to be confirmed by a molecular test.

In this review, after a brief description of the general aspects of the Ebola viruses, we give an overview of the characteristics of molecular tests, rapid diagnostic tests (RDT)s and tests for differential diagnosis, highlighting the aspects concerning the sensitivity and specificity of the single assays.

## General aspects

Ebolaviruses belong to the Monogavirales order, the *Filoviridae* family and the *ebolavirus* genus (7). Among the 12 distinct filoviruses of ebolavirus genus, five infect humans: Ebola virus (EBOV), Reston virus (RESTV), Sudan virus (SUDV), Bundibugyo virus (BDBV), and Tai Forest virus (TAFV). According to the WHO International Classification of Diseases Revision11 (ICD-11) of 2018, there are two major subcategories of filovirus disease (FVD): Ebola disease caused by BDBV, EBOV, SUDV, or TAFV, and Marburg disease caused by MARV or RAVV (belonging to the Marburg genus). Ebola virus disease (EVD) is defined as a disease only caused by EBOV (8).

Since the discovery of filovirus in 1976, 32 ebolavirus outbreaks (excluding at least five laboratory-acquired infections) have been recorded in or exported from Africa, which have been characterized by extremely high case-fatality rates (CFR) ranging from 25% to 90%. The mortality primarily depends on the strain of Ebola virus that causes the outbreak (9).

**Figure 1:**
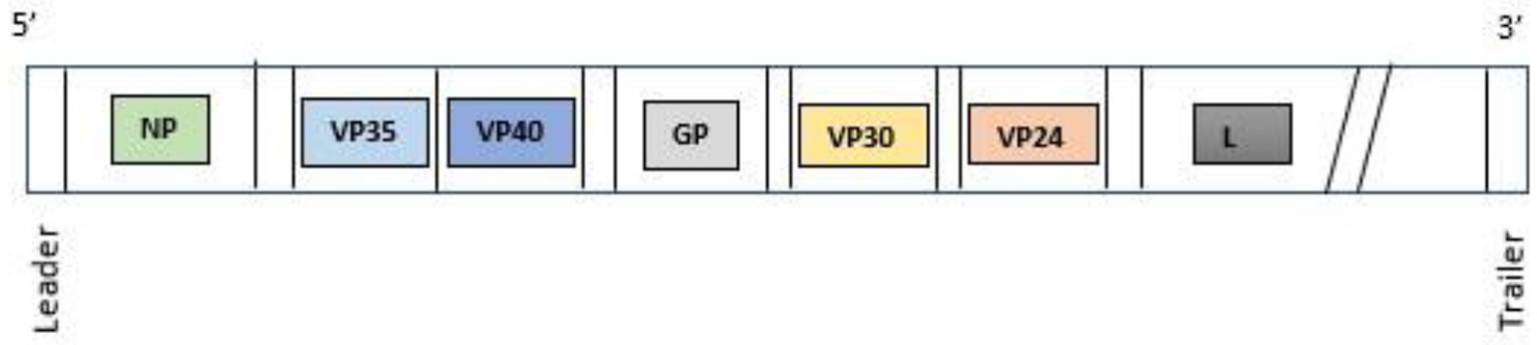
Ebolavirus genome. NP, nucleoprotein; VP35, Ebola viral protein 35; VP40, Ebola viral protein 40; GP, glycoprotein; VP30, Ebola viral protein 30; VP24, Ebola viral protein 24; L, RNA-dependent RNA polymerase.

Ebolavirus has a filamentous, non-segmented negative-sense genome of 19 kb in length, which encodes seven genes and nine proteins (Figure 1). The nine proteins encoded by the EBOV genome are: Glycoprotein (GP), Nucleoprotein (NP), RNA-dependent RNA polymerase (L) and four viral particles: VPs24, 30, 35, and 40. Moreover, two soluble forms of GP: sGP, and small sGP are produced by RNA editing. The GP is the surface protein able to interact with cellular receptors: a sialoglycoprotein receptor on hepatocytes, the folate receptor α on epithelial cells, C-type lectins, present in dendritic cells (DC), macrophages, and endothelial cells. VP40 is a matrix protein that is essential for structural integrity conservation and is located within the viral membrane and is important for filovirus budding. Vp40 is also a matrix protein that influences nucleocapsid formation. NP is an essential component of the nucleocapsid but is also able to activate the replication and transcription of RNA genome. VP30 is a transcriptional activator, while VP35 is a polymerase cofactor. Thus, four proteins: NP, VP35, VP30, and L are associated with the viral RNA genome. Two proteins, VP24 and VP35, antagonize the typeI I IFN responses favoring innate immune evasion (10, 11). Humans can be infected by EBOV through contaminated body fluid, or contaminated fomites, tissue and also through sexual intercourse (12, 13). The bats, *Mops condylurus, Epomops franqueti, Hypsignathus monstrosus, Myonycteris torquatus*, represent the main hosts and reservoir of EBOV and they can transmit the virus to humans (14).

EBOV can be present in breast milk, saliva, urine, semen, cerebrospinal fluid, tears, skin swabs, stool, cerebrospinal fluid, blood and blood derivates, and amniotic fluid. The virus can be transmitted transplacentally and also lead to fetal death due to placental inefficiency (15, 16).

Case fatality rates for each ebolavirus are 33.65 ± 8.38% (BDBV), 43.92 ± 0.7% (EBOV) and 53.72 ± 4.456 (SUDV), which is an average CFR of ~40–50% in the general population (7).

The mean incubation period of EBOV is 6.22 ± 1.57 days for all routes of exposure (17).

During the first phase of infection, the dissemination of virus is promoted by the mononuclear phagocytes and dendritic cells, which represent the primary EBOV targets.

The abundant interleukin production, especially IL-1β, Il-6, and IL-8, and tumor necrosis factor (TNF) in EBOV infected cells are probably the cause of lymphocyte death and linked to disseminated intravascular coagulation, and multiple organ dysfunction syndrome, which is typical of the last phase of EVD.

The clinical manifestations of EVD are similar to those of other pathogens (malaria, typhus, yellow fever): malaise, myalgia, rash, infection; 1–3 days after disease onset, patients develop a non-specific febrile illness, anorexia, arthralgia followed by gastrointestinal symptoms characterized by diarrhea, nausea, vomiting. Generally, the EBOV viral load increases concomitantly with the severity of clinical manifestations. A viral load >10 million genome copies/mL in blood suggests a poor prognosis (18). Higher lethality has also been reported in patients that are co-infected with plasmodium falciparum and plasmodia of other species (19, 20). The late phase is characterized by tissue hypoperfusion and vascular leakage, multiple organ dysfunction syndrome, including hemorrhage from the gastrointestinal tract and alteration of kidney functionality, which is characterized by an abnormal concentration of sodium and potassium and oliguria. The absence of proper hydration can be considered a cause of increased mortality rate in the West Africa outbreak (2014–2016) (21).

The presence of manifestations other than hemorrhage had favored the use of the term Ebola virus disease (EVD) instead of Ebola hemorrhagic fever (22). Rare pauci-asymptomatic infection of EVD has been reported in literature (23). EBOV has also been isolated in the cerebrospinal fluid of EVD infected subjects, suggesting EBOV is a direct agent of meningoencephalitis in EVD (24). In survivors, long term ailments such as psychosis, hepatitis, uveitis have been observed (25).

As previously said, the early recognition of Ebola cases is critical for infection control, since its spread occurs through person-to-person transmission. Individuals can be classified as Persons Under Investigation (PUI) or Confirmed Cases (26).

### Persons under investigation (PUI)

Individuals can be classified as PUI if they have signs and symptoms consistent with EVD. An epidemiological risk factor is to be considered as such 21 days (i.e., the incubation period) before the onset of symptoms.

### Confirmed case

Laboratory-confirmed diagnostic evidence of EVD (i.e., through molecular and/or serologic testing).^2^

As described above, the widest and longest outbreak originated in Guinea (West Africa). It spread to Sierra Leone and Liberia and crossed into Europe and the United States becoming an epidemic.

In addition, an outbreak in the Ituri, Nord-Kivu and Sud-Kivu Province of the Democratic Republic of Congo is the second largest outbreak in terms of the number of cases and deaths, with 3,317 infections and 2,287 deaths (2018–2020) (27). In September 2022, Ugandan health authorities declared an outbreak of Ebola disease, caused by the Sudan virus, in the Mubende district of central Uganda. This is the first Ebola disease outbreak caused by Sudan virus (SUDV) in Uganda since 2012. As of 22 November 2022, a cumulative number of 141 confirmed and 55 probable cases had been reported.^3^ Outbreaks in rural villages without any healthcare infrastructure that is able to ensure rapid diagnosis and appropriate treatment have often favored the spread of infections and a greater mortality rate.

## Management and diagnosis of ebolavirus

EBOV is categorized as a high-hazard pathogen that is handled at Biosafety Level 4, thus it should be managed in Level 4 facilities (BSL4) (28). These laboratories have the following main characteristics: high efficiency particulate air (HEPA) filtered, air-handling systems with negative pressure, all waste material is inactivated and there is a chemical shower for worker decontamination. Staff are highly skilled and trained for years before working at this level of containment. Moreover, mobile diagnostic laboratories could represent a facility to carry out a rapid diagnosis. In a mobile laboratory, there are three separate areas, which include places for inactivation and extraction, reagent preparation, and quantitative reverse-transcription polymerase chain reactions (RT-PCR). In the first area (High Risk area) the samples are inactivated, another area is present for reagent preparation and RT-PCR execution, which is considered a Low Risk area, and a third area is used as office space, with locker rooms and a shower room. To ensure the proper disposal of biohazard waste, there is a medical incinerator capable of temperatures above 100°C. The main advantages of a mobile laboratory are: centralization of diagnostics, rapid result delivery, while the main disadvantages regard the fact it is difficult to obtain a negative pressure environment (29).

### Sample management

Samples should be sent to a lab with facility level 4 in appropriate packaging (UN2814 [category A] or UN3373 [category B] depending on the risk assessment) and using a preapproved courier service.^4^ Literature data report that Ebola viremia could be negative in the early phase of disease. For this reason, the test for Ebola virus should be repeated after 72 h if the first result is negative in subjects with clinical symptoms of EVD or who had strict (close) contact with EVD-positive people.

WHO recommendations for venopuncture, in cases of suspected EBOV or MARV, state that blood should be collected in EDTA tubes with a minimum volume of 5 ml.^5^ The WHO guidelines further state that blood samples can be stored for up to 24 h at room temperature, or at 0–5°C for up to a week. For periods longer than a week, the sample should be stored at −20°C or −70°C (avoiding thaw cycles).^6^

### Ebola virus inactivation

Commonly used disinfectants include 3% Lysol, 5% MicroChem Plus, and 0.5% Hypochrorite solutions. AVL buffer has been determined to not fully inactivate filoviruses, and the addition of ethanol in the procedure is necessary to ensure full inactivation of samples (30, 31).

## Reverse transcription–polymerase chain reaction (RT-PCR)

Thanks to its specificity and sensitivity, RT-PCR is considered the Gold Standard in Ebola diagnosis. It can be qualitative (RT-PCR) or quantitative RT-PCR. In both cases, viral RNA must be purified by extraction steps. Most of the methods for RNA extraction are based on chaotropic methods, which use prevalently guanidium-isothiocyanate and a column with glass fiber filters or magnetic beads for RNA isolation (32). The reverse transcriptase step can be carried out separately from PCR or in a “one-step method”, where all the reagents necessary for reverse transcription and amplification of cDNA are located in the same tube. The primers used in PCR must target the conserved regions of a virus. There are websites available that can support or facilitate this choice. For example, an MRPrimerV database contains a collection of 152, 380,247 high quality primer pairs for the detection of 1818 RNA viruses including Ebola viruses. Primer pairs and probes for RT-PCR can be found for the detection of 7,144 viral coding sequences (CDSs) in this database. This facility offers the possibility to choose primers both for SYBR Green and TaqMan protocols (33).

PCR products are checked in ethidium bromide gel by electrophoresis. Even though this method is inexpensive, it is rarely used because it is time consuming, and a second PCR round (nested PCR) is often required to improve the sensitivity and specificity. Moreover, separate rooms are required for RNA extraction, PCR reagent preparation and gel electrophoresis.

In RT-PCR, forward and reverse oligo primers are used. Since many fluorophores are available in RealTime RT-PCR, several pathogens can be detected simultaneously. The most used probes are TaqMan probes, which are also named 5'hydrolysis probes (34–36) and minor groove-binding probes (MGB). These probes are shorter than TaqMan probes and minor groove probes added at the 3' end makes the interaction between the oligo probes and DNA target more stable. A reaction is considered positive when an exponential increase in the fluorescent signal is observed, while it is considered negative when there is no signal linked to the probe of the viral target, but there is a signal in the corresponding probe of the internal control (IC). In each protocol, allowing RNA quantification, reference standards are used. Generally, *in vitro* transcribed RNA is generated for the target gene and internal control by cloning PCR products into a plasmid vector such as the pCRII TA cloning (Invitrogen). After cloning, the DNA is transcribed into RNA by T7 polymerase. The RNA solution is quantified and serial dilutions are made to obtain a standard curve. The RNA dilution series is amplified in parallel with the patient samples to be quantified. The concentrations of the standard solution are entered into the real-time instrument (LightCycler, Rotorgene, TaqMan, QuantStudio) sample sheet and used for the absolute quantification of the viral target.

Another approach for RNA quantification is SYBR Green I. SYBR Green I dye binds to the minor groove of double stranded DNA. After binding to DNA, the fluorescence of the dye is greatly enhanced and therefore the increase of the fluorescence during the PCR corresponds to the increased amounts of the dsDNA that is amplified. Crucial for an application that uses SYBR Green I is the amplification of the sequence of interest with no specific products. Fluorescence is acquired each cycle during real-time PCR amplification. As the PCR product accumulates, the fluorescence signal increases. The limit of this method is a potential amplification of unspecific byproducts that could interfere with the target amplification, which makes the quantification of the RNA target less reliable (37).

Ct values can vary with each assay platform used for amplification and detection, but the following guidelines can usually be used for the evaluation of the results: Ct values of less than 36.1 indicate a positive reaction, Ct between 36.1 and 39.9 are indeterminate results and require additional testing, and Ct values above 40 indicate negative reactions. Generally, two independent targets are required to consider a sample positive. Patients with negative results, but which have suspected clinical symptoms or contact with Ebola positive subjects, should be retested after 24–48 hours to confirm the negative result. Patients with indeterminate results should also be retested after 24–48 hours (31). To improve the sensitivity and specificity, a dual target assay was carried out for Zaire Ebola virus (EBOV) reaching a sensitivity of 0.4 copies/μL (0.382 copies/μL). The dual target assay was transferred in a GenExpert Flex-03 open cartridge, demonstrating a LOD at 0.75 copies/ μL. The real time for the NP region (nt1007-1086) and GP region (nt 6348-6459) showed higher sensitivity in comparison with the Real time RT-PCR for the L region and Altona RealStar assay. The specificity was assessed with non-related viruses belonging to Filovirus, Alphavirus, Flavivirus, Nairovirus, and Phlebovirus genera. No cross reactivity was observed (38).

Moreover, a PoC microfluidic, real-time, fluorescent-based, continous-flow reverse transcription was developed for the Makona variant Ebola virus. A well conserved region of 120 bp of L gene was chosen as a target. The LOD was 10 copies/μL, which was achieved in 30 minutes (39). In contrast to standard RT-PCR, reverse transcription-loop mediated isothermal amplification (RT-LAMP) does not require high precision thermal cyclers since it is conducted at one temperature. RT-LAMP is less influenced by inhibitors found in the blood and can be used directly with clinical samples. One limit is the inability to multiplex. In a comparative study with RT-PCR, LAMP showed a sensitivity of 97% and positive results are available within 25 minutes (40). Another study on RT-LAMP, which is specific for ZEBOV, demonstrated that RT-LAMP was 10–1,000 fold more sensitive than TaqMan real-time and conventional RT-PCR respectively, reaching a sensitivity of 1 copy/reaction (41).

In order to give an overview of molecular assays for Ebola diagnosis, we show in Table 1 both the main commercial, WHO- and/or Federal Drug Administration (FDA)-approved assays and some in-house methods for virus detection. These molecular assays can be performed on samples of whole blood and its derivatives (plasma and serum), as well as on saliva or urine samples (44, 47, 49, 51, 52, 54). The most widely used targets for detecting the various species of Ebola virus are represented by the gene for the NP nucleoprotein, followed by the L gene. Less represented are assays based on GP and VP40 as targets. Conversely, in four RDTs, VP40 is the main target for molecular tests (Table 2).

**Table 1:** Molecular diagnostic test for Ebola virus.

**Test**	**Test type**	**Virus detected**	**Target gene**	**Biological matrix**	**Reference assay**	**Sensitivity**	**Specificity**	**References**
RealStar Ebolavirus RT-PCR kit (Altona Diagnostics)^**^	rRT–PCR with fluorescent dye-labeled probes to detect PCR amplicons	ZEBOV, BDBV, RESTV, SUDV, TAFV, MARV	L	Plasma	RT-PCR by Panning et al. (36)	LOD values corresponding to 1.9 copies/μL (EBOV Gabon 2003), 1.1 copies/μL (EBOV 2014/ Gueckedou-C05), 6.7 copies/μL (SUDV Gulu), 1.8 copies/μL (BDBV)^*^	100% for ZEBOV. The specificity of the assay was validated with major hemorrhagic fever virus and hepatitis viruses	(42)
Gene Xpert Ebola (Cepheid)^**^	rRT–PCR with fluorescent signal from probes for quality control	ZEBOV	NP	Whole blood and oral fluids	RT-PCR by Trombley et al. (43)	Whole blood: LOD 232.4 copies/mL	Whole blood: 99.5% Oral fluid: 100% Calculated using RT-PCR as benchmark	(44)
FilmArray NGDS BT-E (BioFire)^**^	Fluorescent nested multiplex RT-PCR	ZEBOV	NP	Whole blood, plasma and serum	ND	Whole blood: 10^4^PFU/mL^*^	No cross-reactivity with other Ebola virus or Marburg virus	FilmArray NGDS BT-E Assay Instructions for Use (45)
FilmArray Biothreat-E (BioFire)^**^	Fluorescent nested multiplex RT-PCR	ZEBOV	L	Whole blood and urine	RealStar Ebolavirus RT-PCR kit (Altona Diagnostics) and RT-PCR by Weidmann et al. (46)	Whole blood: 95.7% ^*^	Whole blood: 100% Calculated using RT-PCR as benchmark	(47)
FilmArray Biothreat-E (BioFire)^**^	Fluorescent nested multiplex RT-PCR	ZEBOV	L	Whole blood	Zaire Ebola virus serial dilution	LOD: 600,000 PFU/mL^*^	ND	(48)
Idylla Ebola virus triage test (Biocartis)^**^	rRT–PCR with fluorescent reporter dyes generated upon amplification of cDNA	ZEBOV and SUDV	GP	Whole blood and urine	EZ1 RT-PCR and RT-PCR by Trombley et al. (43)	Whole blood: LOD of 465 PFU/mL for ZEBOV and 324 PFU/mL for SUDV^*^	Whole blood: 100% for ZEBOV and SUDV no cross-reactivity with relevant hemorrhagic fever pathogens	(49)
LightMix Ebola Zaire TIB MolBio with Lightcycler (Roche)^**^	rRT–PCR with fluorescent reporter dye detected at each PCR cycle	ZEBOV	L	Whole blood	ND	Whole blood: LOD: 4,781 PFU/mL^*^	Whole blood: 100% for ZEBOV Benchmark was not specified	(50)
Ebola real-time RT-PCR kit (Liferiver Bio-tech)^**^	Fluorescent rRT–PCR	ZEBOV, BDBV, RESTV, SUDV, TAFV	NP	Serum, oral fluid and urine	RealStar Ebola virus RT-PCR kit (Altona Diagnostics)	Whole blood: LOD: 23.9 copies/reaction^*^	ND	WHO Emergency Use Assessment and Listing Procedure for EVD IVDs PUBLIC REPORT, 2019 (51).
EBOV VP40 real-time RT-PCR (in house method)	rRT–PCR with fluorescent dye-labeled probes to detect PCR amplicons	ZEBOV, SUDV	VP40	Whole blood, serum, plasma and urine	ND	whole blood: LOD of 20–60 TCID50/mL for ZEBOV Mayinga 1976, ZEBOV Kikwit 1995, ZEBOV Gabon 2002^*^	whole blood: 100% for ZEBOV no cross-reactivity with relevant hemorrhagic fever pathogens	CDC 2016. Ebola virus VP40 real-time RT-PCR assay: instructions for use. Atlanta, GA (52).
EZ1 test (DOD) (in house method)	Real-time TaqMan RT-PCR with fluorescent reporter dye detected at each PCR cycle	ZEBOV	GP	Whole blood and plasma	ND	Whole blood: LOD: 5,000 PFU/mL for Ebola Zaire Mayinga virus^*^	100%; no cross-reactivity with other ebola-viruses or Marburg-viruses	Naval Medical Research Center for The U.S. Department of Defense 2014. Ebola Zaire (EZ1) rRT-PCR (TaqMan) Assay: instructions for use (53).
Ebola virus NP real-time RT-PCR (in house method)	rRT–PCR with fluorescent reporter dye detected at each PCR cycle	ZEBOV	NP	Whole blood, serum, plasma and urine	ND	Whole blood and urine: LOD 6 x 10^3^ TCID50/mL^*^	Whole blood urine: 100% for ZEBOV no cross-reactivity with relevant hemorrhagic fever pathogens	CDC 2016. Ebola virus NP real-time RT-PCR assay: instructions for use. Atlanta, GA (54).

^*^The sensitivity of tests was calculated using reference assay as benchmark, references (42, 44, 47, 49, 51), or to the PFU/mL of the virus, references (45, 48, 50, 52–54). ^**^ Approved by the WHO and/or FDA.

BDBV, Bundibugyo virus; CDC, US Centers for Disease Control and Prevention; DOD, US Department of Defense; GP, glycoprotein; L, viral polymerase; LOD, limit of detection; MARV, Marburgvirus; ND, not determined; NP, nucleoprotein; PFU, plaque-forming units; RT-PCR, PCR with reverse transcription; RESTV, Reston Ebolavirus; SUDV, Sudan virus; TAFV, Tai Forest Ebolavirus; TCID50, 50% tissue culture infective dose (concentration at which 50% of cultured cells are infected with a diluted solution of viral bulk); VP40, viral protein 40; ZEBOV, Ebola virus Zaire.

**Table 2:** Rapid diagnostic test for Ebola virus.

**Test**	**Test type**	**Virus detected**	**Target**	**Biological matrix**	**Reference assay**	**Sensitivity**	**Specificity**	**References**
ReEBOV RDT (Corgenix, USA)^**^	Immuno-chromatographic assay	ZEBOV, SUDV, BDBV	VP40	Whole EDTA blood, serum, plasma	RealStar Filovirus Screen RT-PCR kit (Altona Diagnostics, Germany)	Plasma: 93.2%^*^	Whole EDTA blood: 98%. Plasma: 80.3%. Calculated using rRT-PCR as benchmark	(55)
SD Q Line Ebola Zaire Ag test (SD Biosensor, Korea)^**^	Immuno-precipitation lateral flow assay	ZEBOV	GP, NP, VP40	Plasma, serum and whole blood	RealStar Filovirus Screen RT-PCR kit (Altona Diagnostics, Germany)	Plasma: 84.50%^*^	Whole EDTA blood: 100%. Plasma: 99.0%. Calculated using rRT-PCR as benchmark	(55)
OraQuick Ebola Rapid Antigen Test (OraSure Technologies, USA)^**^	Immuno-chromatographic lateral flow assay	BDBV, ZEBOV, SUDV:	VP40	Oral fluid and whole blood	RT-PCR GeneXpert Ebola Test (Cepheid, USA)	Whole blood: 57.4%^*^	Whole blood: 98.3%. Calculated using rRT-PCR as benchmark	(56)
Dual Path Platform (DPP) Ebola antigen system (Chembio, USA)^**^	Immuno-chromatographic lateral flow assay	ZEBOV	VP40	Venous whole EDTA blood, venous plasma, serum and capillary fingerstick	RT-PCR by Trombley et al. (43)	Serum: 77.10%^*^	Serum: 91.7% cross reactivity with malaria and other relevant HF pathogens	(57)
QuickNavi-Ebola (Denka, Seiken, Tokyo, Japan)^**^	Immuno-chromatographic assay	ZEBOV, BDBV, TAFV:	NP	Whole blood and serum	RT-PCR GeneXpert Ebola Test (Cepheid, USA)	Whole blood: 85%^*^	Whole blood: 99.8%. Calculated using r Gene Expert RT-PCR as benchmark	(58)
E-ZYSCREEN RDT (CEA, Saclay, France)	Lateral flow immunoassay	ZEBOV	GP	Whole blood and serum	RealStar Filovirus Screen RT-PCR kit (Altona Diagnostics, Germany) and RT-PCR by Weidmann et al. (46)	Whole blood: 65.3%^*^. Serum: 74.5%^*^	Whole blood: 98.8%. Serum: 100%. No cross reactivity with major hemorrhagic fever virus	(59)
DEDIATEST Ebola (Senova, Germany)	Lateral flow immunoassay	ZEBOV	VP40	Serum, throat swab, whole EDTA blood, plasma	RealStar Filovirus Screen RT-PCR kit (Altona Diagnostics, Germany)	Plasma: 79.5%^*^	Whole EDTA blood: 100%. Plasma: 84.3%. Calculated using Altona rRT-PCR as benchmark	(55)
One step Ebola test (Intec, China).	Lateral flow immunoassay	ZEBOV	ND	whole EDTA blood, plasma	RealStar Filovirus Screen RT-PCR kit (Altona Diagnostics, Germany)	Plasma: 98.4%^*^	Whole EDTA blood: 95%. Plasma: 80.2%. Calculated using Altona rRT-PCR as benchmark	(55)
Ebola Ag K-SeT (Coris BioConcept, Gembloux, Belgium)	Immuno-chromatographic lateral flow assay	ZEBOV	VP40	Whole blood, plasma and serum	RealStar Filovirus Screen RT-PCR kit (Altona Diagnostics, Germany)	Plasma: 88.6%^*^	Plasma: 98.1%. The specificity of the assay was validated with malaria, HIV, hepatitis viruses, Zika Virus, DENV3, and EBV	(60)
NMRC EBOV LFI (Naval Medical Research Center for EBOV, Bethesda, USA)	Lateral flow immunoassay	SUDV, TAFV, RESTV	GP	Plasma, serum, oral swab	RT-PCR by Trombley et al. (43)	Plasma: 87.8%^*^. Oral swab: 88.9%^*^	Plasma: 97.5%. Oral swab: 96.1%. Calculated using rRT-PCR as benchmark	(61)

^*^The sensitivity of the tests was calculated using reference assay as benchmark, references (55–61). ^**^Current commercially available tests.

BDBV, Bundibugyo virus; DENV3, Dengue serotype 3; EBV, Epstein-Barr virus; GP, glycoprotein; HIV, Human Immunodeficiency Virus; NP, nucleoprotein; RESTV, Reston Ebola virus; SUDV, Sudan virus; TAFV, Tai Forest Ebola virus; VP40, viral protein 40; ZEBOV, Ebola virus Zaire; HF, Hemorrhagic Fever.

Furthermore, it has been observed that Zaire Ebola species is the most detected in both commercial and home-made tests, followed by Sudan Ebola species.

As shown in Table 1, the specificity in both home-made assays and commercial kits appears to be 100%, with no particular cross-reactivity among the various Ebolaviruses or Marburgvirus species. The molecular tests reported in Table 1 show a good sensitivity both in commercial and in-house assays.

Among the RT-PCRs that target the L gene, the Altona assay RealStar Ebolavirus RT-PCR 1.0 kit, which is the first Ebola molecular diagnostic test approved by WHO, appears to be one of the most sensitive. The analytical sensitivity was determined by testing dilutions of *in vitro* transcript for SUDV Gulu, MARV Popp and Musoke, BDBV, and EBOV Gabon 2003 and EBOV Gueckedou-C05. The Filovirus Screen kit achieved the following LOD values: 1.9 RNA copies/μL of RNA eluate (95% [CI], 1.1–3.3) for EBOV Gabon 2003, 1.1 (95% CI, 1.0–1.2) for EBOV 2014/Gueckedou-C05, 6.7 (95% CI, 4.2–24) for SUDV Gulu, 1.1 (95% CI, 22–11) for MARV Popp, 4.2 (95% CI, 1.9–18) for MARV Musoke, and 1.8 (95% CI, 1.6–2.1) for BDBV (52). This sensitivity of the RealStar Ebolavirus RT-PCR kit was calculated by comparing it against the Panning 2007 reference assay (36). The LightMix Ebola Zaire TIB MolBio with Lightcycler (Roche) showed a sensitivity in whole blood indicated as LOD of 4,781 PFU/mL (50). The Biothreat-E FilmArray (BioFire) had an LOD of 600,000 PFU/mL (48). For the NP gene, Gene Xpert Ebola (Cepheid) was found to be the assay with the best sensitivity with a limit of detection of 232 copies/mL (44). Another commercial NP gene kit with good sensitivity is FilmArray NGDS BT-E (BioFire). It showed an LOD of 10,000 PFU/mL in whole blood (45). The Idylla Ebolavirus triage test (Biocartis) is a commercial test based on the detection of a region of the GP gene of Ebola Zaire and Ebola Sudan virus. It has an LOD of 465 PFU/mL and 324 PFU/mL for ZEBOV and SUDV respectively. The only in-house RT-PCR assay targeting the VP40 gene is EBOV VP40 real-time RT-PCR developed by the US CDC. Using whole blood as a biological matrix, the LOD is 20 and 60 TCID50/ml for EBOV and SUDV respectively (52) (Table 1). A portable real-time PCR machines such as Biomeme have the potential to bring real-time PCR diagnostics as point of care (Biomeme Inch, Philadelphia, PA, United States).

### Sequencing

Sequencing is not often used in the molecular diagnostics of EBOV because of the presence of low-resource laboratories in geographical areas where outbreaks occur. Sanger sequencing is mainly used for molecular epidemiology investigation. Moreover, during the 2014–2016 EBOV outbreak, a sequence device, MinION (Oxford nanopore Technologies), was used in Guinea for the sequencing and analysis of EBOV samples. For the general surveillance of circulating viruses in a region, next generation sequencing has been proposed (62, 63).

## Rapid diagnostic test (RDT)

The difficulty in accessing an early and fast diagnosis that guarantees the efficient contact tracing and isolation of EVD positive patients limits the control of epidemics. It has been hypothesized that the use of RDTs with a specificity and sensitivity of 99% could reduce the number of cases in the Sierra Leone epidemic by 42% (64).

Current molecular diagnostic methods such as the polymerase chain reaction require trained personnel and laboratory infrastructures, which hinder diagnostics at the point of need, particularly in outbreak settings and frequently the samples should be sent to a reference center to be analyzed with a delay in the availability of results. Point of care rapid diagnostic tests substantially reduce these delays.

In November 2014, the WHO issued a call for rapid, sensitive, safe and simple Ebola diagnostic tests strictly related to ASSURED indications: minimal laboratory facility, no cold chain and can be performed with a capillary blood sample collected through a finger prick, with a test result in minutes rather than days (65).

The ASSURED criteria of the WHO for PoC devices are: affordable, sensitive, selective, user-friendly, rapid, equipment free, and deliverable (to end users). Furthermore, in filovirus outbreak situations, PoC devices could play a key role in the triage of patients that arrive at a clinic with fever. A key aspect of PoC devices is that there is minimal sample handling and potentially pathogenic material does not require transport to distant sites, thereby improving the diagnostic turnaround time. REASSURED represents an update of ASSURED indications. REASSURED recommends a fast transmission of test results, an increase of sample collection and a strengthening of network data sharing (66).

FDA expands upon the user friendly term to include minimal required training, no precise measurements, no user interpretations, and no user intervention steps (67). According to the WHO, rapid diagnostic test indications for Ebola virus should have a clinical sensitivity of > 95% and a specificity of 99% (64).

Therefore, a rapid diagnostic test that is used at a PoC should be compliant with ASSURED criteria.

Several laboratories have developed new RDTs (Table 2). Commercially available tests include ReEBOV, SD Zaire Ag, OraQuick, Ebola Antigen System and QuickNavi-Ebola. The ReEBOV test received an emergency use authorization from the FDA and the WHO during the 2013–2016 Ebola outbreak. However, in 2018, the FDA revoked this authorization when the new manufacturer (Zalgen Laboratories) that had acquired the company failed to reproduce the claimed test accuracy of ReEBOV (68). These RDTs can be performed on capillary blood, whole blood with EDTA, plasma, serum or even saliva (56).

Most of the tests listed in Table 2 are designed to detect VP40 protein (55–57, 60). However, other tests are directed against NP or GP proteins of the virus (53, 55, 59).

For VP40 protein, the sensitivity in plasma of DEDIATEST Ebola (Senova, Germany) is 79.53% and for Ebola Ag K-SeT (Coris BioConcept, Gembloux, Belgium) it is 88.6%, while the specificity is 84.3% and 98.1% respectively considering the Altona Real Star Filovirus screen RT-PCR Kit as a reference assay (55, 60). The OraQuick Ebola Rapid Antigen Test (OraSure Technologies, USA) has a sensitivity of 57.4% and a specificity of 98.3%, compared with the RT-PCR GeneXpert Ebola reference test (Cepheid, USA) in whole blood (56). The Dual Path Platform (DPP) Ebola antigen system (Chembio, USA) has a sensitivity of 77.16% and a specificity of 91.77% in serum (57). For GP protein, the sensitivity of the SD Q Line Ebola Zaire Ag test (SD Biosensor, Korea) is 84.5% while the specificity is 99.0% in plasma and 100% in whole EDTA blood, using the Altona molecular assay as reference test (55).

In whole blood, E-ZYSCREEN RDT (CEA, Saclay, France) has a sensitivity and specificity of 65.3 and 98.8% respectively, while in serum samples, the sensitivity and specificity are 74.5 and 100% respectively considering the Altona molecular assay as reference test (59).

Concerning the NMRC EBOV LFI (Naval Medical Research Center for EBOV, Bethesda, USA) (53), different sensitivity has been observed in plasma (87.8%) and oral swabs (88.9%). The specificity was 97.5% using the plasma as template, while it was 96.1% when an oral swab was used as template. The RT-PCR by Trombley was used as reference test (61).

The QuickNavi-Ebola test is based one NP protein (Denka, Seiken, Tokyo, Japan). This choice is linked to several NP properties: (1) strong antigenicity, (2) multiple antibody binding sites that facilitate an increase in sensitivity, (3) antigen epitope sharing among different species of Ebola viruses (58). The sensitivity and specificity in whole blood of QuickNavi-Ebola is 85 and 99.8% respectively, using the RT-PCR GeneXpert Ebola Test as a reference molecular assay (Cepheid, USA) (58) (Table 2). From these data, it can be seen that there is no particular statistical difference between the various targets, although GP seems to be less sensitive than the other genes (69). Although some of the RDTs show appreciable accuracy, to date, no commercial rapid diagnostic assays have reached the sensitivity and specificity indicated by WHO. Tests with better performance will need to undergo further field studies during future outbreaks (68). Moreover, improved monoclonal antibodies and/or aptamers and the use of multiple antigens have the potential to improve the sensitivity and specificity of RDTs.

## Differential diagnosis

The symptoms observed in EVD patients are similar to those linked to other hemorrhagic fever infections: fever, myalgia, chills, malaise.

The non-specific symptoms EVD pose a major problem to triage and isolation efforts at Ebola Treatment Centers (ETCs). Viral hemorrhagic fevers (VHF) and malaria have overlapping clinical characteristics making differential diagnosis a challenge. A new immunoassay has been developed called surface-enhanced Raman scattering (SERS), which at the same time detects antigens from Ebola, Lassa, and malaria within a single blood sample. Results are provided quickly (< 30 min) for each sample. To test the performance of this assay, 190 Ebola positive clinical samples, 163 malaria positive samples and 233 negative controls were used. The results showed a sensitivity of 90.0% and specificity of 97.9% in Ebola detection. In malaria detection, the sensitivity and specificity were 100.0 and 99.6%, respectively. These results indicate the potential of the SERS technology as an important tool for outbreak management in low-resource settings (70).

Since a low number of probe sets can be included in real-time PCR, this platform is considered to be of limited use in differential diagnosis.

An oligonucleotide microarray is a high throughput technology that seems to be accurate, speedy, and low-cost. It has been used for disease diagnosis, pathogenic microorganism detection, gene expression analysis, and single-nucleotide polymorphism (SNP) detection. A study reported the establishment of an oligonucleotide microarray method for the simultaneous identification of 16 pathogens associated with hemorrhagic fever, including Zaire ebolavirus, Sudan ebolavirus, Marburg virus (MARV), LASFV, Junin virus (JUNV), Machupo virus (MACV), Rift Valley fever virus (RVFV), Crimean-Congo hemorrhagic fever virus (CCHFV), malaria parasite (MP), hantavirus (HV), severe fever with thrombocytopenia syndrome virus (SFTSV), dengue virus (DENV), YFV, Chikungunya virus (CHIKV), influenza A virus (FluA), and influenza B virus (FluB). The signal was obtained following a chemiluminescence approach. The sensitivity of microarray analysis for Marburg and Ebola virus was 10^3^ copies/μL (71). This low sensitivity and an amplification step render the microarray not suitable in diagnostic activity.

TaqMan array card (TAC) uses quantitative reverse transcription-PCR (qRT-PCR) for the simultaneous detection of 15 viruses (Chikungunya, Crimean-Congo hemorrhagic fever (CCHF) virus, dengue, EBOV, Bundinbugyo virus, SUDV, hantaviruses [Hantaan and Seoul], Hepatitis E, MARV, Nipah virus, O'nyong-nyoung virus, Rift Valley fever virus, West Nile virus, and YFV), 8 bacteria (Bartonella spp, Brucella spp, Coxiella Burneti, Leptospira spp, Rickettsia spp, *Salmonella enterica* and Salmonella enterica serovar Typhy and Yersinya pestis), and 3 protozoa (Leishmania spp, Plasmodium spp, and *Trypanosoma brucei*) of particular relevance to sub-Saharan Africa. TaqMan array cards are stable at 4°C for two years and could be shipped at room temperature. TAC exhibited an overall sensitivity of 88% and a specificity of 99%. This TaqMan array card can be used in field settings as a rapid screen for outbreak investigation or for the surveillance of pathogens, including Ebola virus. The lower limit of detection was estimated to be 10^4^ copies/mL with a serum sample (72). However, the sensitivity of PCR assays is not normally an issue in diagnosing cases of filovirus infection. Virus titers in Ebola virus infections can reach 10^10^ RNA copies/mL when collected near death and 10^5^ RNA copies /mL at the onset of symptoms (34).

## Enzyme-linked immunosorbent assays (ELISA)

Traditional serological diagnostics of Ebola virus-infected patients is usually carried out using an antigen ELISA assay (73). In the early stages of infection, the body produces an IgM response but the anti-Ebola IgM titre decreases after 1 month post-onset of infection, eventually becoming undetectable (74).

The people with EVD that do not survive often die before mounting a proper humoral immune response. This suggests that antibody-based detection systems for Ebola may not be useful in Ebola diagnosis (69).

To detect filovirus species-specific antibodies, a new ELISA test was developed, using a secreted form of the transmembrane GPs of different Ebola species. However, the mouse antisera IgG antibodies showed a cross reactivity among several filoviruses (75).

Limited data are available to assess the sensitivity and specificity of the ELISA test. In one study carried out in convalescent-phase sera from different outbreaks, poorly IgG cross reactivity was observed among Reston, Bundibugyo, Zaire and Sudan Ebola virus (76). This cross reactivity was less extensive regarding IgM (76). These findings suggest that different vaccines are required to protect against different Ebola viruses. A new ELISA assay was carried out to differentiate antibody vaccine-induced with Ebola and those linked to natural infection. The authors used peptides of EBOV VP40, VP35 and NP of 181 human sera collected from healthy controls, EBOV vaccinated, and EBOV-infected survivors. This new “EBOV-Detect” ELISA demonstrated >94% specificity and 96% sensitivity for the diagnosis of EBOV infection.

Serological assays could be used for epidemiology and the surveillance of EBOV infections during and after outbreaks, especially in countries with structured Ebola vaccination campaigns (77). The virus neutralization test should be considered as the gold standard to identify individuals who have been infected with EBOV.

To date, there are no commercial assays to detect IgM and IgG antibodies for Ebola virus.

## Immunofluorescent assay

An indirect immunofluorescent assay (IFA) using virus-infected Vero cells can be used for the detection of antibodies of Ebola viruses in BSL4. The samples can be tested at a fixed dilution 1:20, both for IgM and IgG detection. Moreover, the positive samples can be tested at limiting dilution. The IgG and IgM titres are reported as the reciprocal of the highest dilution with positive fluorescence (WHO) (78). Furthermore, an immunofluorescent method based on NP recombinant protein was developed. Hela cells were transfected with baculovirus expressing EBOV NP. These cells were used to detect anti-Ebola IgG. This method is very sensitive and represents an alternative to procedures that can be only carried out in BSL4 facilities (79). To date, there are no commercial tests for IFA Ebola IgG and IgM detection.

## Viral isolation

In the past, cultures of live agents were considered the gold standard for viral detection, but currently PCR and next generation sequencing are becoming more and more relevant in diagnostics.

Ebola viral isolation can be performed from the patient's serum using Vero E6 cell lines and several days (range from 3- to 6 days) are required to detect the cytopathic effect. This assay is not used for primary diagnosis because it is less sensitive than PCR and requires a level 4 laboratory (BSL4 facilities).

However, viral isolation is useful for virulence or pathogenesis studies (80).

Moreover, through viral isolation, one can obtain a consistent viral titer that can be used to sequence the whole genome or for molecular epidemiology studies.

## Electron microscopy

Electron microscopy (EM) can be used to visualize the virus particles in specimens directly from clinical materials or viral culture. This technique was used for the diagnosis of filovirus infections in past decades (69, 81–85).

Viral structures can be visualized in serum samples from Ebola positive patients or in supernatants since initial passage infected cell cultures (69). The immuno-EM method is a feasible method to perform for the detection of a filovirus that is serologically related to EBOV (81). An indirect immuno-electron microscopy method has been successfully applied for the identification of Ebola Reston (EBOV-R) particles in serum and tissue culture fluid specimens. The immuno-EM method was also used to differentiate antigenically different filoviruses (83).

## Discussion

The data reported in this review underline how a rapid diagnosis is essential for efficient contact tracing to contain the spread of the infection and prevent modest outbreaks from becoming a health emergency. In fact, it has been calculated that if 60% of patients are rapidly diagnosed within one day of the onset of symptoms, the attack rate curve drops from 80 to 0% (86). RT-PCR-based methods/assays are conventional diagnostics methods, although the sensitivities of molecular biology tests are difficult to compare with each other given that there is no International Reference Standard.

Nevertheless, in remote and rural settings, this conventional diagnostics-based tool is difficult to perform and the biological samples should be send to a Reference laboratory, which causes delays in reaching a proper diagnosis (87). A long wait often pushes medical personnel to mis-administer drugs against the febrile state assuming that it is due to a bacterial infection, with the administration of antibiotics or antimalarials with a consequential worse outcome due to inappropriate treatment (88, 89). This delayed correct diagnosis of infectious disease also contributes to the spread of infection.

Furthermore, co-infections with more than one pathogen is frequent, and differentiating between them is essential for successful disease management. In this context, PoC tests have arisen as a rapid and accurate diagnostic approach in the global health field as suggested by the WHO in November 2014. However, as demonstrated in this review, the rapid molecular tests do not reach the sensitivity and specificity indicated by the WHO. Furthermore, many tests have been developed for a specific species, very often for EBOV (Table 2), the virus responsible for the 2014–2015 epidemic that originated in Guinea and which rapidly spread in Liberia and Sierra Leone.

The specificity is often below 90%. For example, in a real life study where the WHO-approved ORAQUICK rapid antigen test was used, there were 15% false positives (90).

On the other hand, experimental rapid tests based on the detection of nucleic acids on a microfluidic platform are being developed (39). They show excellent sensitivity and provide results in 30–50 min (a longer execution time allows for greater sensitivity to be achieved). However, they are not yet available on the market and therefore we do not know in real life their sensitivity and specificity.

Another aspect that should be considered is the availability of rapid diagnostic tests. Despite their vital importance for effective patient management, they are hardly available and the costs are high, so much so that their purchase becomes difficult (91).

Furthermore, in a work/survey carried out by Cnop et al. (91), it was highlighted that many rapid diagnostic tests were no longer produced by the Company after the end of the epidemic and therefore they cannot be used for surveillance activities in geographical zones where Ebola is endemic. This aspect should be corrected so that the use of PoCs is always made possible in all health facilities, even and above all those in rural areas, which do not have molecular biology laboratories and highly specialized personnel to perform molecular tests (92).

## Author contributions

AB, DL, and AG contributed to the conception of the work, the drafting of the paper, critical revision of the review, and final approval of the version to be published. All authors contributed to the article and approved the submitted version.

## Footnotes

^1^ https://apps.who.int/mediacentre/news/ebola/18-november-2014-diagnostics/en/index.html

^2^ https://www.cdc.gov/vhf/ebola/clinicians/evaluating-patients/index.htm

^3^ https://africacdc.org/disease-outbreak/outbreak-brief-8-sudan-ebola-virus-disease-evd-in-uganda/

^4^ https://assets.publishing.service.gov.uk/

^5^ http://www.who.int/csr/resources/publications/ebola/blood-collect/en/

^6^ https://www.cdc.gov/vhf/ebola/laboratory-personnel/specimens.html
